# Analyzing the Impact of Ambient Temperature Indicators on Transformer Life in Different Regions of Chinese Mainland

**DOI:** 10.1155/2013/125896

**Published:** 2013-06-13

**Authors:** Cui-fen Bai, Wen-Sheng Gao, Tong Liu

**Affiliations:** ^1^Department of Electrical Engineering, Tsinghua University, Beijing 100084, China; ^2^Electric Power Research Institute, CSG, Guangzhou, Guangdong 510080, China

## Abstract

Regression analysis is applied to quantitatively analyze the impact of different ambient temperature characteristics on the transformer life at different locations of Chinese mainland. 200 typical locations in Chinese mainland are selected for the study. They are specially divided into six regions so that the subsequent analysis can be done in a regional context. For each region, the local historical ambient temperature and load data are provided as inputs variables of the life consumption model in IEEE Std. C57.91-1995 to estimate the transformer life at every location. Five ambient temperature indicators related to the transformer life are involved into the partial least squares regression to describe their impact on the transformer life. According to a contribution measurement criterion of partial least squares regression, three indicators are conclusively found to be the most important factors influencing the transformer life, and an explicit expression is provided to describe the relationship between the indicators and the transformer life for every region. The analysis result is applicable to the area where the temperature characteristics are similar to Chinese mainland, and the expressions obtained can be applied to the other locations that are not included in this paper if these three indicators are known.

## 1. Introduction

The life of a transformer is defined as the life of the paper insulation [1]. The paper insulation deteriorates with several factors, primarily including temperature, moisture content, and oxygen content. In all these factors, temperature has a major impact because the contribution of moisture or oxygen to the insulation deterioration can be minimized with modern oil preservation systems [2]. 

The temperature of a transformer, often considered as hot spot temperature (HST), is primarily controlled by ambient temperature and load, where ambient temperature is the dominated factor. On one hand, some types of load are directly affected by ambient temperature, such as the cooling load in summer: the higher the ambient temperature is, the greater the load will be. On the other hand, the load capability of a transformer is generally governed by ambient temperature. Transformers have to operate under the prescribed limit of HST, and ambient temperature is an uncontrollable factor in the influential factors of HST (ambient temperature and load); therefore, if the ambient temperature is high, the load capability of a transformer is always low, and vice versa. From these two aspects, it can be seen that ambient temperature is an important factor in estimating the transformer life.

There are some literatures that studied the impact of an increase of ambient temperature on transformer life. Reference [3] used a standardized engineering approach according to IEEE Standard C57.91 [4] to preliminarily assess the impact of increased ambient temperature on transformer loss of life at five locations in the USA. For an assumed 4°C rise in ambient temperature during 1900–2100, the predicted loss of life in the interval from 1990 to 2045 rises approximately 32% at a relatively warm location and 8% at a relatively cold location. Reference [5] applied an improved model of top oil temperature rise differing from the IEEE C57.91 model to assess the impact of ambient temperature change on transformer life, based on seven transformers and four loading test beds specified in IEEE C57.115. With the assumption of temperature rise of 3.5°C in 100 years, a probabilistic model for ambient temperature rise was performed to conclude that a reduction in the life of a transformer was about 3–6 years for the case studied, and there was a marked difference in the mean life of a transformer for several different loading conditions.

These literatures focus on the impact of ambient temperature rise on transformer life, and the impact of different temperature characteristics on transformer life at different locations is not included. Actually, the ambient temperature characteristics of one location have a great impact on the local transformer life. For example, the transformer life at a warmer area is shorter than that at a colder area. Furthermore, there are many indicators portraying temperature characteristics in meteorology, and the key issue related to transformer life prediction and power system operation is which indicators are most important for the transformer life. 

This paper focuses on quantitatively analyzing the impact of different ambient temperature characteristics on transformer life at different locations of Chinese mainland and attempts to find the most important temperature indicators for transformer life estimation based on regression analysis. Chinese mainland is selected for study due to its vast territory and diverse climates. In practical situations, difference in latitude, longitude, or altitude results in complex temperature characteristics; different temperature characteristics cause different values of transformer life. The life consumption model in IEEE Std. C57.91-1995 [2] is employed to estimate different values of transformer life at 200 typical locations of Chinese mainland. These locations are specially divided into six regions. For each region, the local historical temperature and load data are provided as inputs variables of the life consumption model to estimate the transformer life at every location. Then, the partial least squares regression (PLSR) method is applied to construct the regression between the transformer life and five temperature indicators. Finally, based on a criterion to measure the contribution of temperature indicators in PLSR, three indicators are considered the most important factors and involved in the regression analysis for every region. The relationship between the transformer life and these three temperature indicators is formulated with a simple and acceptable equation for every region, and the equations can be used for life estimation at the locations that are not included in this paper.

## 2. Transformer Life Estimation at Different Locations

This section presents the calculation process of the transformer life at different locations of Chinese mainland based on the life consumption model in IEEE Std. C57.91-1995.

### 2.1. Introductions to IEEE Life Consumption Model

The extensively employed life consumption model in IEEE Std. C57.91-1995 [2] is applied in this paper. In the model, the transformer life is established as a function of hot spot temperature (noted as *θ*_*H*_) which is computed by ambient temperature and load. Then, *θ*_*H*_ is provided as an input variable to acquire the thermal aging acceleration factor *F*_AA_ [2]:

(1) $$\begin{array}{c} F_{\text{AA}}=\exp\left(\frac{15000}{383}-\frac{15000}{\theta_{H}+273}\right) \end{array}$$

when *θ*_*H*_ is at the reference temperature 110°C *F*_AA_ is equal to 1. Based on this definition, the loss of life in a given time period is proposed as [2]

(2) $$\begin{array}{c} F_{\text{EQA}}=\frac{\sum_{n=1}^{N}F_{{\text{AA}}_{n}}\Delta t_{n}}{\sum_{n=1}^{N}\Delta t_{n}}, \end{array}$$

(3) $$\begin{array}{c} \%\;\;\text{Loss}\;\;\text{of life}=\frac{F_{\text{EQA}}\times t\times 100}{\text{Normal insulation life}}, \end{array}$$

where *F*_EQA_ is the equivalent aging factor in the total time period, *F*_AA_*n*__ is the aging acceleration factor during the time interval Δ*t*_*n*_, *N* is the total number of time intervals, and “Normal insulation life” is the proposed value of transformer life at the reference temperature 110°C.

According to the model, ambient temperature and load are two important root causes to the life consumption. The configuration of these two variables will be proposed subsequently to calculate the transformer life. In addition, to simplify the calculation process of the transformer life, ambient temperature and load are assumed to be annually cyclic so that only a sample annual temperature curve and a sample annual load curve on an hourly basis are needed to calculate the transformer life. With this assumption, the life value obtained for one location may not be very accurate, but it has little effect on the analysis result because the aim of this paper is to compare the life values at different locations, and the hypothesis and predigestion in calculation process for all locations are the same.

### 2.2. Hourly Ambient Temperature Curve

**Figure 1:**
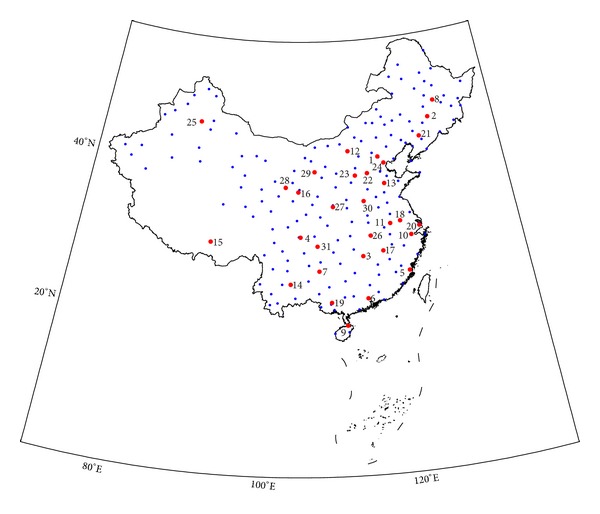
200 meteorological stations in Chinese mainland. The stations in important cities are marked in red.

Ambient temperature is an input variable of the IEEE model. We obtain historical temperature records from the total of 200 meteorological stations for this research [6]. These locations have a variety of temperature and load characteristics so that they can provide a relatively comprehensive description of different climate and load types in Chinese mainland.  Figure 1 illustrates the 200 locations on the map, and the important cities (capital cities, municipalities directly under the central government, or capitals of autonomous region) are labeled specially. The locations cover the latitude range approximately from 19°N to 51°N, longitude range from 76°E to 132°E, and historical temperature records are available from 1951 to 2011 for most meteorological stations. The temperature data includes daily maximum temperature (*T*_max,*d*,*y*_, *d* = 1,2,…, 365, *y* = 1,2,…, 61) and minimum temperature (*T*_min,*d*,*y*_).

To obtain a sample annual temperature curve on an hourly basis for every location, mean daily maximum temperature $\left(\bar{T_{\text{max,}d}}\right)$ and mean daily minimum temperature $\left(\bar{T_{\text{min,}d}}\right)$ are calculated by averaging the data set in 61 years, according to

(4) $$\begin{array}{c} \bar{T_{\text{max,}d}}=\frac{1}{61}\sum_{y=1}^{61}T_{\text{max,}d,y}\;\left(d=1,2,\ldots ,365\right),\\ \bar{T_{\text{min,}d}}=\frac{1}{61}\sum_{y=1}^{61}T_{\text{min,}d,y}\;\left(d=1,2,\ldots ,365\right). \end{array}$$

**Figure 2:**
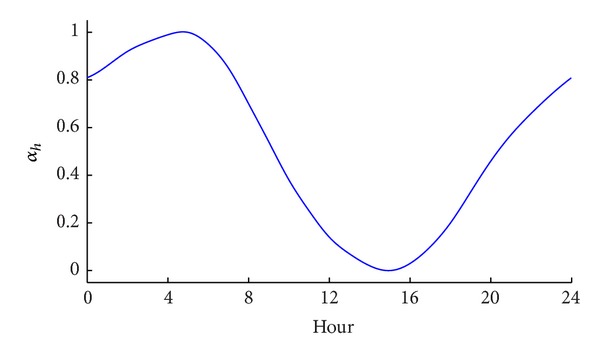
Temperature ratio, a parameter used to obtain the hourly temperature [7].

Then mean daily maximum temperature and mean daily minimum temperature in 365 days of the sample year are acquired. They are used to obtain the hourly temperature curve, according to [7]

(5) $$\begin{array}{c} T_{h,d}=\bar{T_{\text{max,}d}}-\alpha_{h}\left(\bar{T_{\text{max,}d}}-\bar{T_{\text{min,}d}}\right)\\ \left(h=1,2,\ldots ,24,\;\;d=1,2,\ldots ,365\right), \end{array}$$

where *T*_*h*,*d*_ is the temperature at hour *h* in the day *d* and *α*_*h*_ is the temperature ratio shown in Figure 2 [7]. Hence, the hourly temperature curves in the sample year for the 200 locations are formed.

### 2.3. Hourly Load Curve

**Figure 3:**
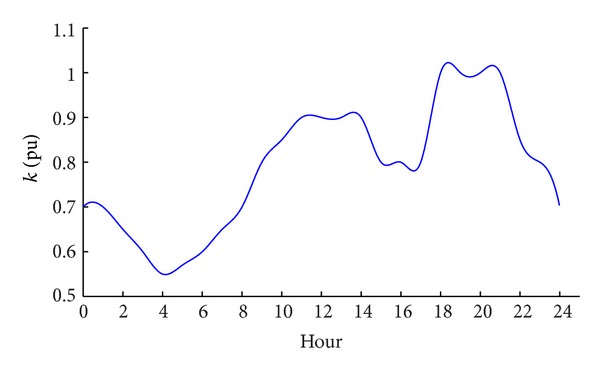
Hourly load variation in a day.

Load is the other input variable of the IEEE model. We set the hourly load curve for every location according to the statistics data [8, 9]. The load repeats both daily and annually, described by hourly and monthly load variation curves. For hourly load variation curves, 200 locations are hypothesized to have the same shape as Figure 3 because the actual difference is not significant.

**Figure 4:**
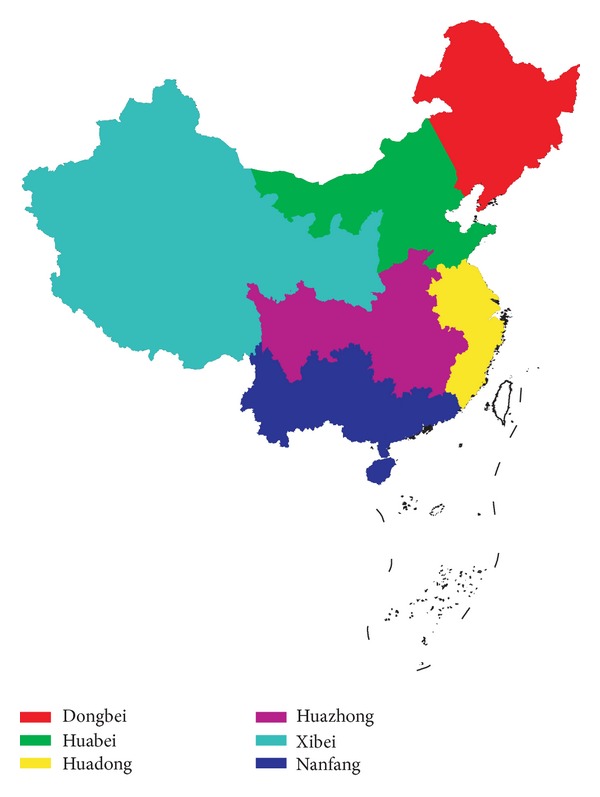
200 locations are divided into six regions.

**Figure 5:**
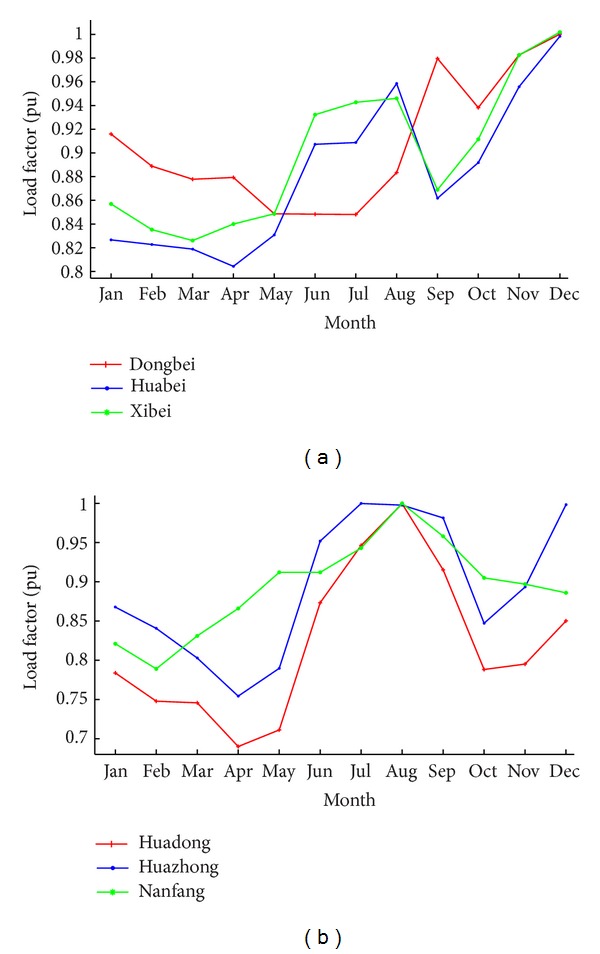
Monthly load variation curves of the six regions [8, 9].

However, the monthly load variation curves are significantly different from location to location due to many influential factors. Based on the load statistics [8, 9], locations are divided into six regions as Figure 4 illustrates, including Dongbei, Huabei, Huadong, Huazhong, Xibei, and Nanfang, and the number of locations included in every region is given in Table 1. The locations in one region have similar load characteristics, and the difference between two regions becomes large. The regional average monthly load variation curves are given in Figure 5 according to [8, 9]. It shows that every region generally has a summer load peak and a winter load peak in one year, but some have larger winter load (Figure 5(a)), and some have larger summer load (Figure 5(b)). Accordingly, the monthly load variation curves can be roughly classified into two categories.

The curves in Figures 3 and 5 are used conjunctionally to form the hourly load curve in the sample year, according to

(6) $$\begin{array}{c} k_{h,m}=\frac{k_{m}}{\bar{k_{h}}}\cdot k_{h},\;h=1,2,\ldots ,24;\;\;m=1,2,\ldots ,12, \end{array}$$

where *k*_*h*,*m*_ is the load factor in hour *h* of the month *m*, *k*_*m*_ is the average monthly load factor in Figure 5, *k*_*h*_ is the hourly load variation in Figure 3, and $\bar{k_{h}}$ is the average load factor in Figure 3. 

As a result, all locations in one region have the same hourly load curve. Such treatment aims to do the analysis in Section 3.

### 2.4. Local Transformer Life Estimation

**Figure 6:**
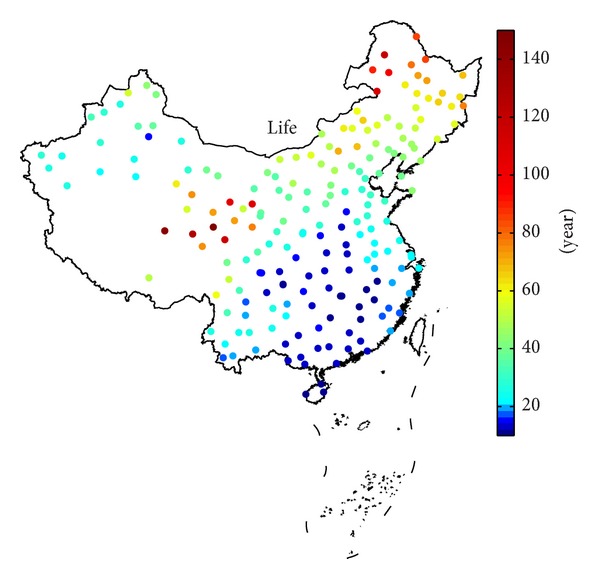
Calculation results of the transformer life values at 200 locations.

The local hourly ambient temperature and load curves in the sample year are inputted into the IEEE life estimation model. The parameters of a sample transformer used are shown in Table 2, most of which are extracted from [10]. The “Normal insulation life” in (3) is set to be 180000 h according to [2]. The calculation results of the transformer life values at all locations are shown in Figure 6. The range for the value of life is about 10.6–149.3 years. It can be seen that the distribution of transformer life has a certain geographical feature, which will be discussed in details in the next section.

## 3. Analyzing the Impact of Ambient Temperature on Transformer Life

Section 2.4 obtained the transformer life values at 200 locations and concluded that the transformer life has a geographical feature. In this section, we will discuss the reason for it by analyzing the temperature and load characteristics. Then, the relationship between ambient temperature indicators and transformer life will be quantified by regression analysis.

### 3.1. The Temperature and Load Characteristics in Chinese Mainland

**Figure 7:**
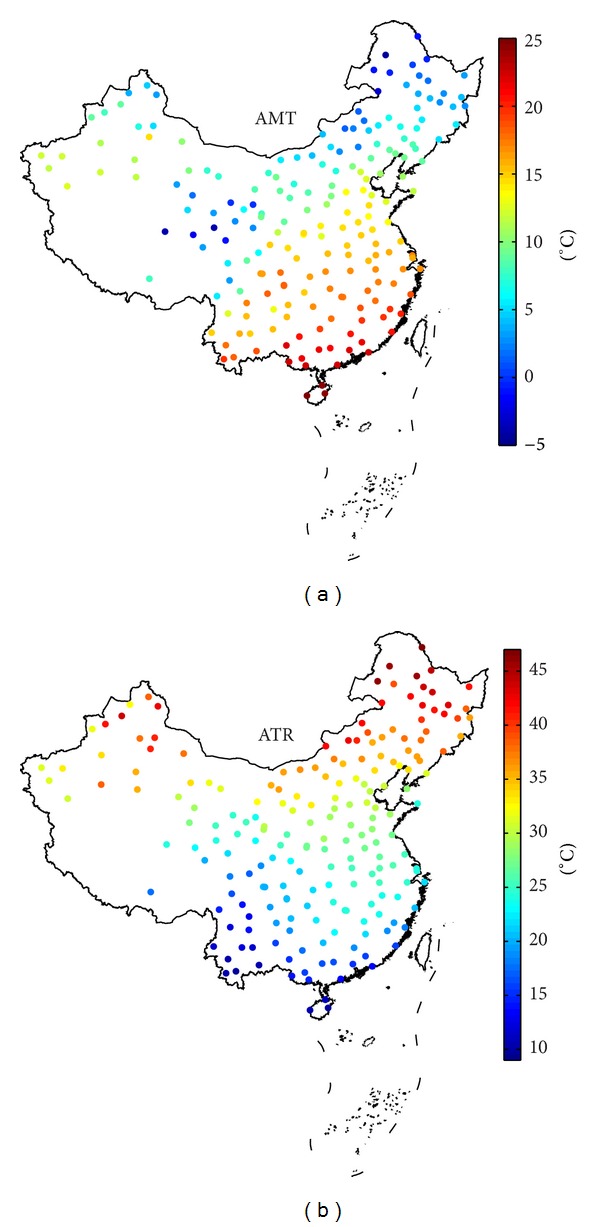
(a) Annual mean temperature at 200 locations; (b) annual temperature range at 200 locations.

Figure 6 shows that the distribution of transformer life has a certain geographical feature, which can be inferred from the geographical feature of temperature and load. After the hourly temperature curve in the sample year is got in Section 2.2, many indicators describing the temperature characteristics can be acquired, such as annual maximum (minimum) temperature and annual mean temperature (AMT).  Figure 7 illustrates the annual mean temperature and annual temperature range (ATR) at the 200 locations to give a general description of the temperature characteristics in Chinese mainland. AMT shows the annual mean temperature level at one location, and ATR is the mean temperature difference between the warmest and coldest months during one year, demonstrating the temperature amplitude variation mostly from summer and winter. ATR can be obtained according to

(7) ATR=max⁡m⁡(1n∑d=1n(124∑h=124Th,d,m))−min⁡m⁡(1n∑d=1n(124∑h=124Th,d,m)),

where *T*_*h*,*d*,*m*_ is the temperature in hour *h* of the day *d* of the month *m* and *n* is the number of days in the month *m*.  Figure 7(a) shows that the locations can be approximately divided into north and south, and it is warmer in south than in north on annual mean temperature level basis.  Figure 7(b) illustrates that the temperature difference from large to small is in the northern area, central area, and southern area. Generally, the temperature levels in the warmest month are similar all across the country, and the differences of ATR primarily come from the coldest month. Therefore, the northern area has the lowest temperature level in the coldest month, and the climate of the southern area is more moderate correspondingly.

For the load characteristics, it can be found from Figures 4 and 5 that North China has larger winter load, and South China has larger summer load. The load has characteristics similar to the temperature. One reason is that the temperature is a primary influential factor of the load. For the regions in Figure 5(a), the cold weather of winter causes a larger heating load in winter compared to the cooling load in summer. This phenomenon is more pronounced in Dongbei compared to the other two in Figure 5(a) due to the significant larger winter load resulting from its extreme cold weather in winter. For the regions in Figure 5(b), their indistinct seasons and mild temperature in all year result in larger cooling load in summer than the heating load in winter.

The discussion above demonstrates the preliminary characteristics of temperature and load distribution in Chinese mainland. Different types of ambient temperature and load will result in different values of life; therefore, the distribution of transformer life also has its characteristics at these locations. In addition, the ambient temperature characteristics largely affect the characteristics of the load. Therefore, the ambient temperature is an extremely important factor influencing the life of a transformer in normal operation.

### 3.2. The Impact of Ambient Temperature on Transformer Life

From Figures 6 and 7, we can find that there are a rough positive correlation between the transformer life and AMT and a rough negative correlation between it with ATR. It is worth mentioning that because all locations in one region have the same hourly load curve in the sample year as discussed in Section 2.3, the differences in transformer life for the locations in one region are considered primarily from the different temperature characteristics. The treatment is a starting point for the analysis of the relationship between temperature characteristics and transformer life. Consequently, the similarities between the life distribution and the temperature can be found, and their relationship can be investigated and analyzed in a regional context.

#### 3.2.1. The Selection of Regression Variables

The regression technique [11], a widely used relationship assessment method, is performed to assess the relationship between transformer life and ambient temperature and find out the significantly influential temperature indicators. The temperature indicators involved in the regression analysis are picked out firstly. AMT is chosen as a representative temperature indicator which reflects the overall temperature level of one location. Generally, it affects the transformer life strongly because the life is consumed greatly when the ambient temperature is high according to the Arrhenius theory [12] (when the ambient temperature is high, the transformer is generally subjected to a heavier load meanwhile which also increases the life consumption). Based on this theory, another two indicators, the mean temperature of the warmest day (MTWD) and the mean temperature of the warmest month (MTWM), are included. Additionally, diurnal temperature range (DTR) and ATR are involved to take account into the temperature range. DTR is defined as the temperature difference between the maximum and the minimum temperatures during the day, which describes the temperature amplitude variation in one day. These five temperature indicators can provide a relatively comprehensive description of the temperature characteristics of one location, and they are noted as  *x*_1_(AMT),  *x*_2_(MTWD), *x*_3_(MTWM),  *x*_4_(DTR), and  *x*_5_(ATR).

**Figure 8:**
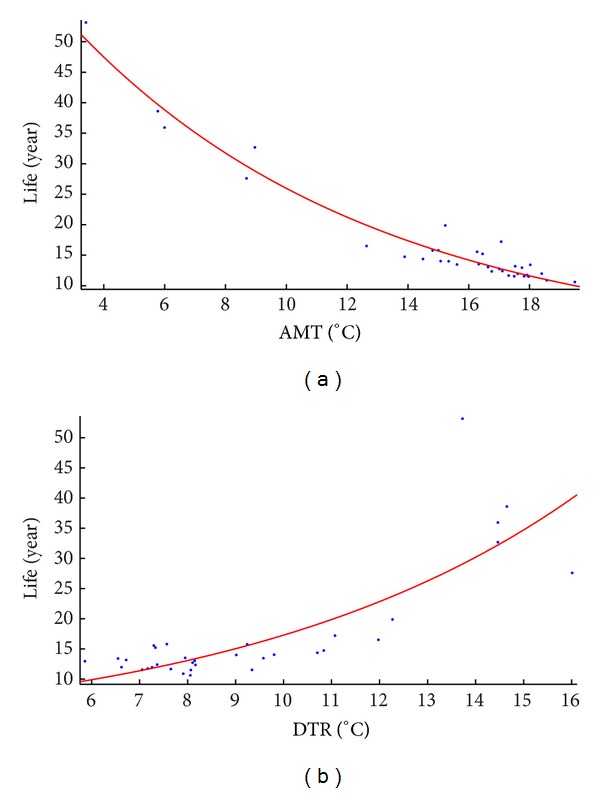
The relationship between transformer life and temperature indicators in Huazhong. (a) Life versus annual mean temperature. (b) Life versus diurnal temperature range.

The dependent variable involved in the analysis is the transformer life. An indicator-life scatterplot can preliminarily describe the relationship between transformer life and temperature indicators. For example, Figure 8 illustrates the relationship for two indicators in Huazhong, respectively, where it is approximately expressed in an exponential form. The similar conclusion can also be drawn for the other five regions. Actually, this fact is associated with the exponential form of (1). The natural logarithm of the variable “transformer life” is taken before the regression analysis so that the multiple linear regression (MLR) can be conducted conveniently. The variable “transformer life” after the natural logarithm, noted as *y*, is considered the dependent variable of the regression analysis.

#### 3.2.2. Partial Least Squares Regression

Correlation coefficient analysis is applied to check the correlation of these variables.  Table 3 shows the result for Huadong as an example. It can be seen that there are strong correlation between *y* and every *x*_*i*_  (*i* = 1, 2,…, 5), which confirms the important influence of temperature indicators on the transformer life.

In addition, there is also a close correlation between every *x*_*i*_, which identifies the multiple correlations among the independent variables. This multicollinearity problem must be paid attention in order to find the most crucial temperature indicators influencing the transformer life and analyze the relationship between the indicators and the transformer life. Under this situation, the classical MLR is hard to employ because it will obtain an unstable or impractical solution [13], and PLSR is a good alternative because it is more robust [13–16].

PLSR combines the functions of principal component analysis and MLR, aiming at analyzing a set of dependent variables from a set of independent variables [15]. It constructs a new set of orthogonal factors extracting from the independent variables, often called latent variables or components, to predict the dependent variables. The latent variables capture most of the information in the independent variables and have the best predict power; therefore, the regression can be constructed by using fewer components than the numbers of independent variables, and the dimensionality of the regression problem is reduced [14, 15]. In addition, because finding the most influential variables on the dependent variables interests many analysts, the variable importance in the projection (VIP) scores obtained by the PLSR has been paid an increasing attention as an important measure of each independent variable [17, 18]. VIP is a criterion to measure the contribution of independent variables to the regression in PLSR, and its analytical definition can be found in [17]. VIP will be applied to measure the importance of each temperature indicator.

#### 3.2.3. The Calculation Result and Discussion

**Figure 9:**
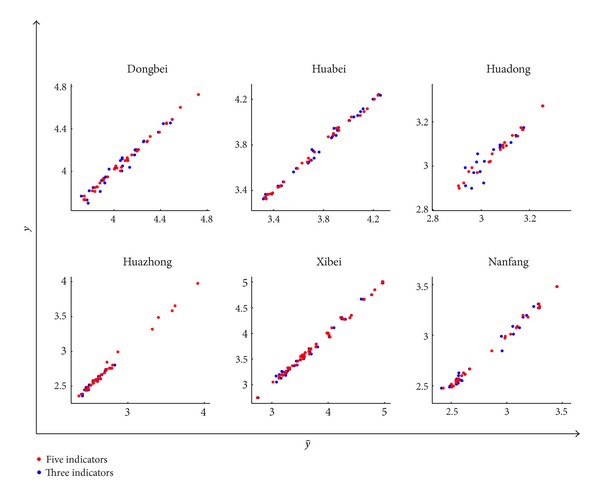
The real value of transformer life (*y*) versus the predicted one ($\hat{y}$).

Based on the PLSR, one can construct an estimation formula of transformer life:

(8) $$\begin{array}{c} \hat{y}=\beta_{0}+\beta_{1}x_{1}+\beta_{2}x_{2}+\beta_{3}x_{3}+\beta_{4}x_{4}+\beta_{5}x_{5}, \end{array}$$

where *β*_*i*_  (*i* = 0,1,…, 5) are the regression coefficients. The red markers in Figure 9 demonstrate that with these five indicators, the predicted value of the transformer life from (8) (horizontal axis) and the actual value of life (vertical axis) are in good agreement, which verifies the correctness of the regression result.

**Figure 10:**
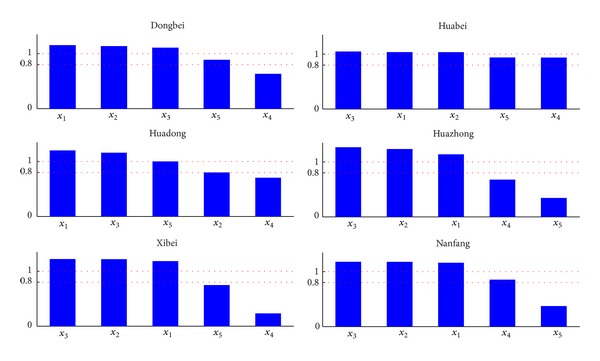
Variable importance in the projection scores of five variables in six regions: *x*_1_(AMT), *x*_2_(MTWD), *x*_3_(MTWM), *x*_4_(DTR), and *x*_5_(ATR).

Figure 10 illustrates the VIP values of the five variables in these six regions. It can be seen that the variables have different important levels in different regions. According to their relevance in explaining *y*, **X** could be classified as follows: VIP > 1.0 (highly influential), 0.8 < VIP < 1.0 (moderately influential), and VIP < 0.8 (less influential) [18]. It can be found that *x*_1_ and *x*_3_ have great influence on the transformer life in every region. In other words, the annual mean temperature and mean temperature of the warmest month are important for the transformer life. For AMT, it is not surprising because transformer life has a strongly positive correlation with it based on the aforementioned discussion. The importance of MTWM implies that the loss of life in the warmest month accounts for a large percentage in the whole year. Accordingly, controlling the load level in the warmest month becomes especially significant in order to postpone the lifetime of the transformer in warm regions.

It can be also seen from Figure 10 that only parts of temperature indicators are strongly related to the transformer life in every region, some indicators are apparently less relevant than the others, and the VIP values of some are smaller than 0.8. Additionally, there are some similarities that *x*_1_, *x*_2_, and *x*_3_ are highly influential in most regions except for Huadong. The difference between Huadong and the other regions may be attributed to the less sample data in Huadong compared to the other regions as shown in Table 1. This result implies that AMT, MTWD, and MTWM of one location affect the life of transformer strongly. In contrast, the temperature difference, either day or year, is less important in the life estimation. Considering the convenience of practical application, we attempt to construct the PLSR with only *x*_1_, *x*_2_, and *x*_3_. The regression coefficients are listed in Table 4, and Figure 9 also gives the regression result with three indicators. It can be seen from Figure 9 that the difference between the result with five indicators and with three indicators is small, and an acceptable accurate level still can be achieved with only *x*_1_, *x*_2_, and *x*_3_. In this case, the values of VIP of these three temperature indicators in every region are almost equal except that the VIP of *x*_2_ is a little smaller than *x*_1_, *x*_3_ in Huadong.

Conclusively, AMT, MTWD, and MTWM are considered the most important indicators influencing the life of transformers in all regions of Chinese mainland. The conclusion is meaningful due to the large span in latitude and longitude and a wide variety of climatic conditions in Chinese mainland, and it is applicable to the area where the temperature characteristics are similar to Chinese mainland. In addition, a unified and common regression model is constructed with these three variables involved for all these six regions in Chinese mainland. It can be applied to the other locations that are not included in this paper if these three variables are known. 

## 4. Conclusion

Ambient temperature is an important factor in estimating transformer life. 200 locations divided into six regions in Chinese mainland are selected to study the impact of various temperature characteristics on transformer life. According to the historical records of ambient temperature and the statistics data of load curves, preliminary characteristics of temperature and load distribution in Chinese mainland are discussed. Different types of ambient temperature and load will result in different values of life. Based on the IEEE life model, the calculated result shows that the transformer life in the 200 locations ranges from 10.6 to 149.3 years.

Additionally, the assumption of the load configuration causes the difference in transformer life at different locations in one region to be considered primarily from the different temperature characteristics at different locations. Consequently, the effect of temperature indicators can be investigated and analyzed in a regional context. 

To quantitatively analyze the impact of different ambient temperature characteristics on transformer life, PLSR is performed with five independent variables (annual mean temperature, mean temperature of the warmest day, mean temperature of the warmest month, diurnal temperature range, and annual temperature range) for every region. These five indicators can provide a comprehensive description of the temperature characteristics and give a well prediction of the transformer life at one location. Furthermore, considering the convenience of practical application, we use VIP as a contribution criterion to assess the importance of the independent variables, and annual mean temperature, mean temperature of the warmest day, and mean temperature of the warmest month are considered the most important indicators influencing the life of transformers for all regions. The conclusion is applicable to the area where the temperature characteristics are similar to Chinese mainland, and the regression model with these three variables can be applied to the other locations that are not included in this paper. 

## Figures and Tables

**Table 1:** The number of locations in every region.

Region	The number of locations
Dongbei	35
Huabei	34
Huadong	19
Huazhong	34
Xibei	49
Nanfang	29

**Table 2:** The parameters of the sample transformer.

Parameter	Value
Δθ_*TO*,*R*_	45°C
Δθ_*H*,*R*_	40°C
*R*	1.0
*τ*_*TO*_	3.5 h
*m*_*w*_	0.8
*n*_o_	1.0

**Table 3:** Correlation coefficient matrix in Huadong.

	*y*	*x*_1_	*x*_2_	*x*_3_	*x*_4_	*x*_5_
*y*	1	−0.9633	−0.9897	−0.9919	0.8493	−0.6177
*x*_1_		1	0.9363	0.9351	−0.8738	0.3976
*x*_2_			1	0.9986	−0.8724	0.6866
*x*_3_				1	−0.8617	0.6946
*x*_4_					1	−0.437
*x*_5_						1

**Table 4:** The regression coefficients.

Region	$\hat{y}=\beta_{0}+\beta_{1}x_{1}+\beta_{2}x_{2}+\beta_{3}x_{3}$			
	*β*_0_	*β*_1_	*β*_2_	*β*_3_
Dongbei	6.6077	−0.0238	−0.0535	−0.0517
Huabei	5.8204	−0.0225	−0.0388	−0.0393
Huadong	5.9147	−0.0197	−0.0366	−0.0520
Huazhong	4.6873	−0.0197	−0.0300	−0.0303
Xibei	5.3413	−0.0251	−0.0312	−0.0312
Nanfang	4.9882	−0.0301	−0.0311	−0.0306
